# Supplementary material for: TRPML1 gating modulation by allosteric mutations and lipids
Source: bioRxiv. 2024 Sep 14:2024.07.04.602033. Originally published 2024 Jul 6. Preprint. [Version 2] doi: 10.1101/2024.07.04.602033 (PMC11245044; doi:10.1101/2024.07.04.602033)
Supplement: Supplement 3 [file NIHPP2024.07.04.602033v2-supplement-3.pdf]

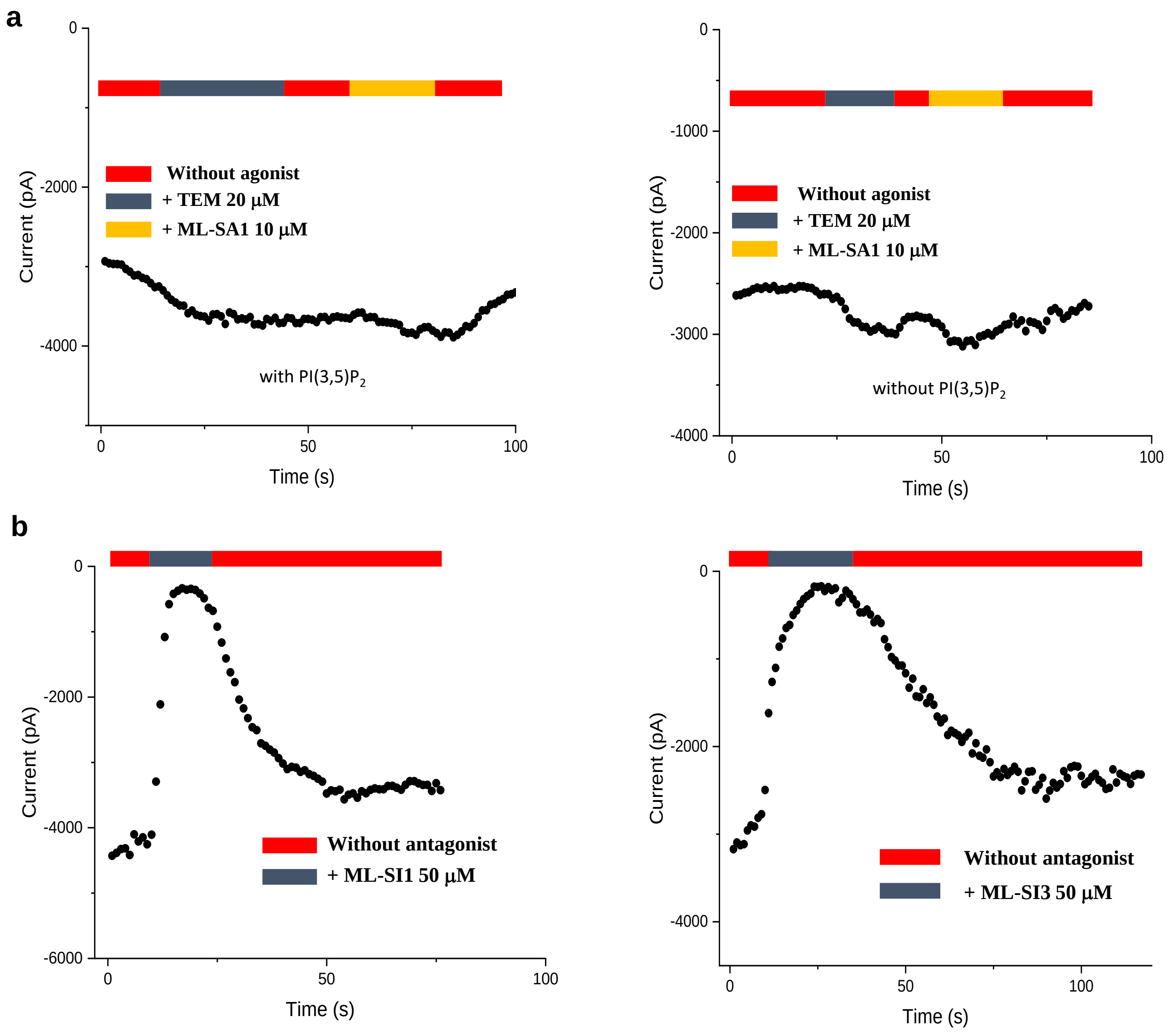

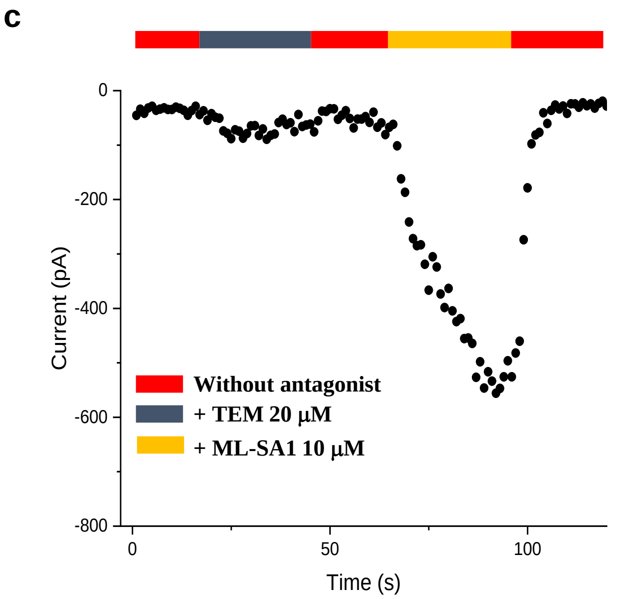

Figure supplement 1: Time course plots of current amplitudes of Y404 mutations recorded at -140mV with symmetrical pH of 7.4. (a) Time course plots of Y404W recorded using patch clamp in whole-cell configuration with (left) or without (right) 100 µM PI(3,5)P_2 in the pipette (cytosolic). Tem or agonist ML-SA1 was introduced in the bath solution (extracellular/luminal). (b) Time course plots of Y404W inhibition by antagonists ML-SI1 (left) and ML-SI3 (right) recorded using patch clamp in whole-cell configuration. The antagonists were introduced in the bath solution (extracellular/luminal). (c) Time course plots of Y404A with 100 µM PI(3,5)P_2 in the pipette (cytosolic). Tem or ML-SA1was introduced in the bath solution (extracellular/luminal).

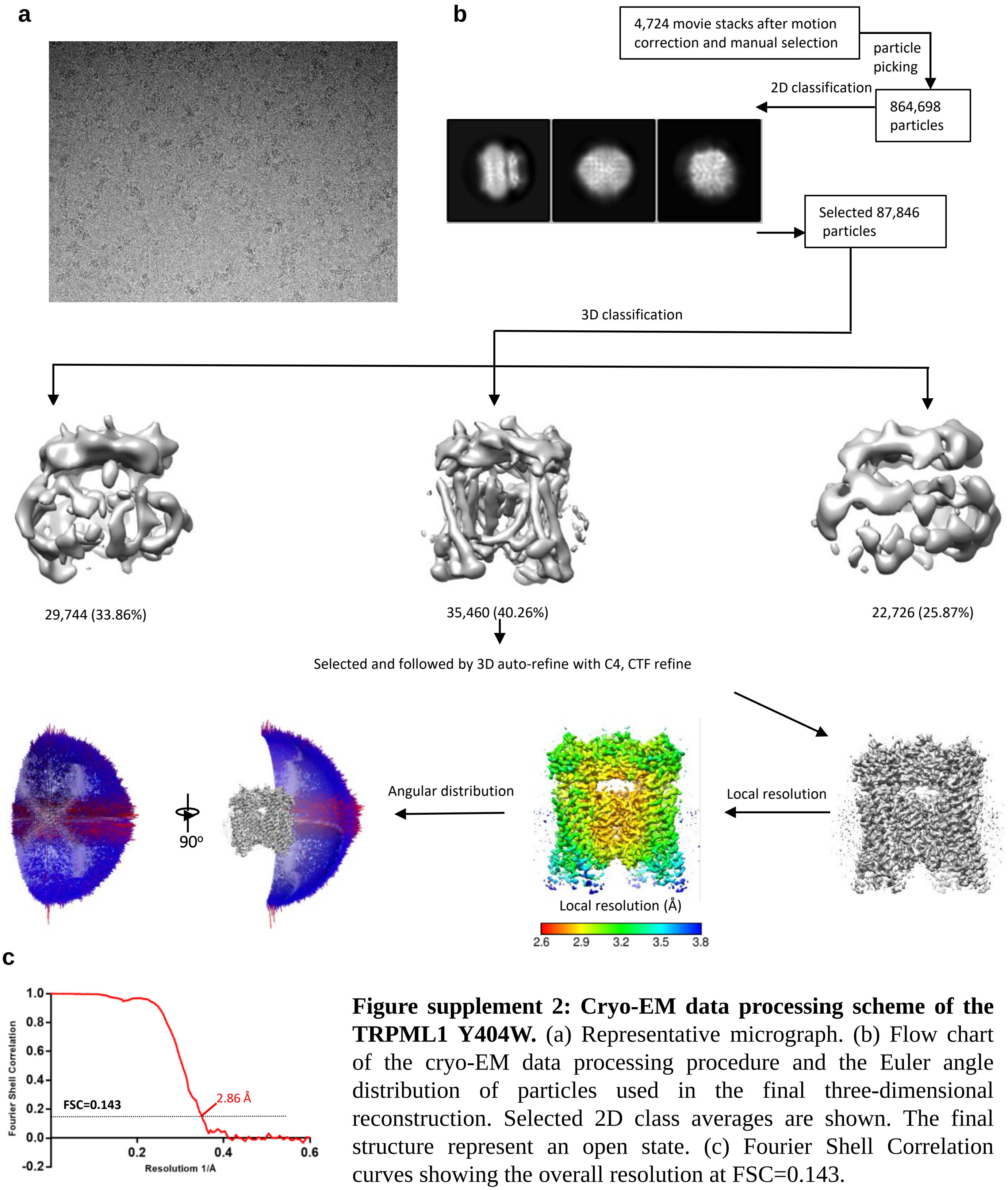

Figure supplement 2: Cryo-EM data processing scheme of the TRPML1 Y404W. (a) Representative micrograph. (b) Flow chart of the cryo-EM data processing procedure and the Euler angle distribution of particles used in the final three-dimensional reconstruction. Selected 2D class averages are shown. The final structure represent an open state. (c) Fourier Shell Correlation curves showing the overall resolution at FSC=0.143.

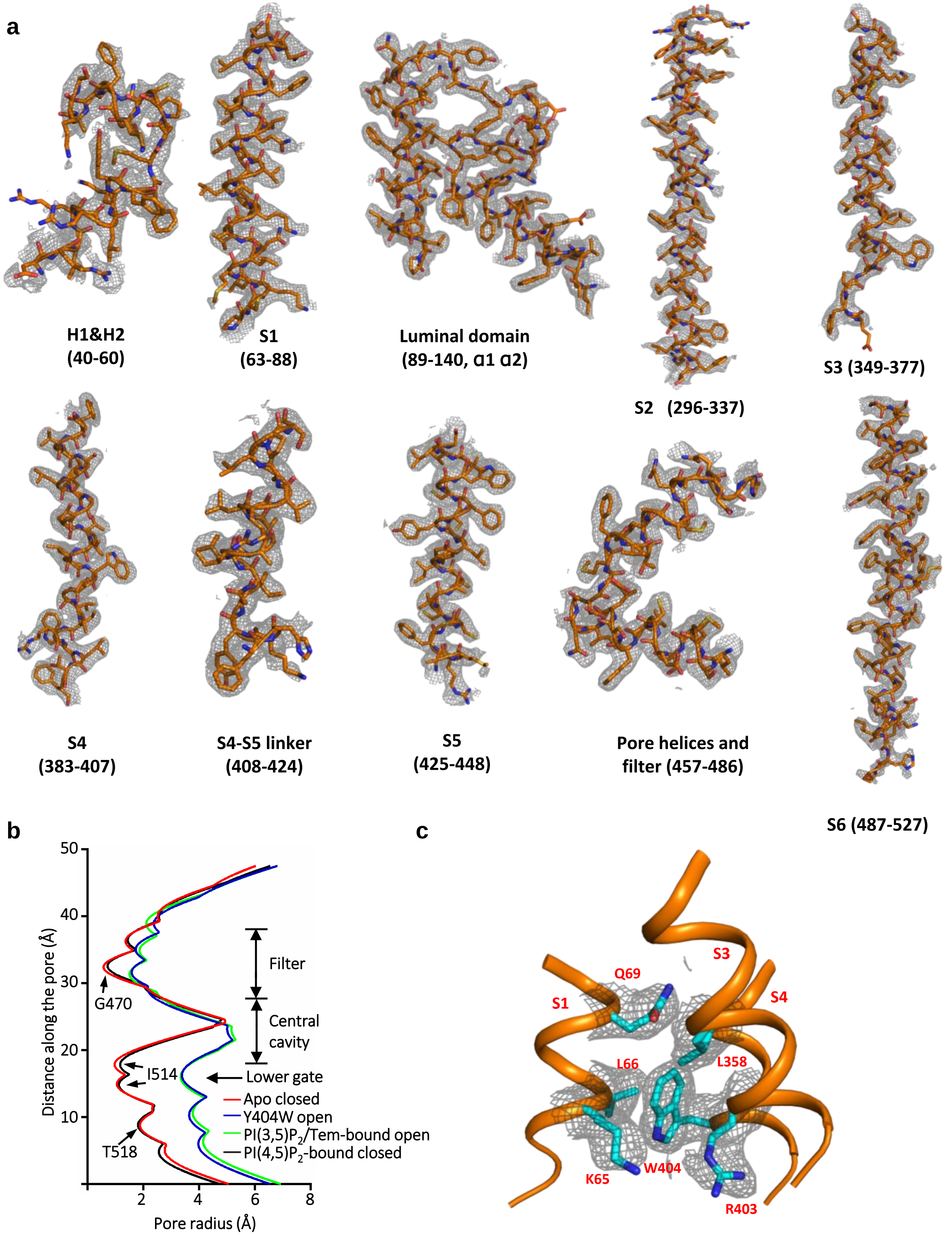

Figure supplement 3: Sample density maps of Y404W and pore radius: (a) Sample density maps of the Y404W open TRPML1 structure contoured at 4 σ. (b) Pore radius along the central axis in the open and closed states. PDB codes for apo closed and PI(3,5)P_2/Tem-bound open are 7SQ8 and 7SQ9, respectively. (c) EM density map surrounding W404 region shown in grey mesh and contoured at 4σ, key W404-interacting residues are shown in cyan. The local resolution of this region is 3.2 Å.

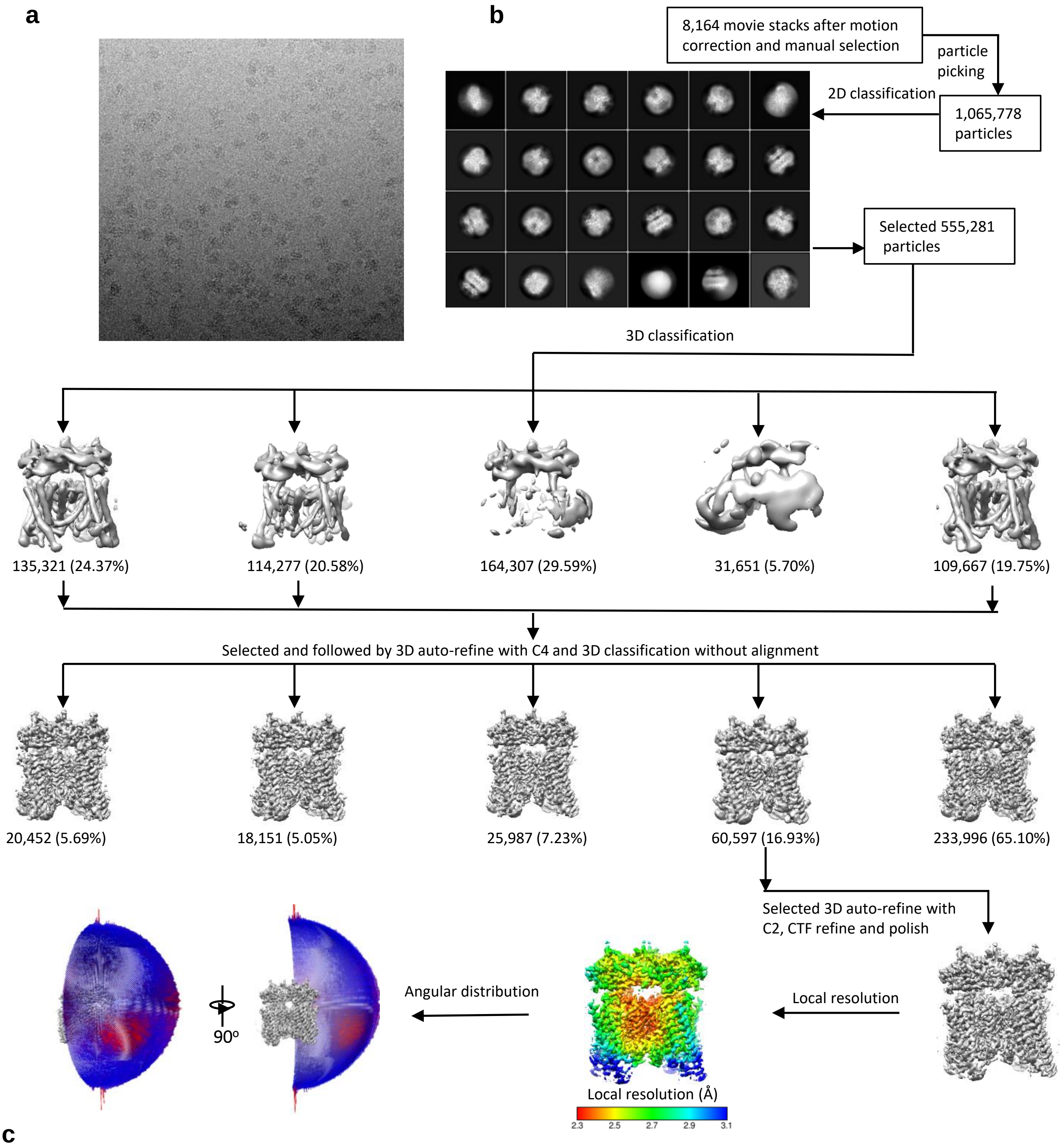

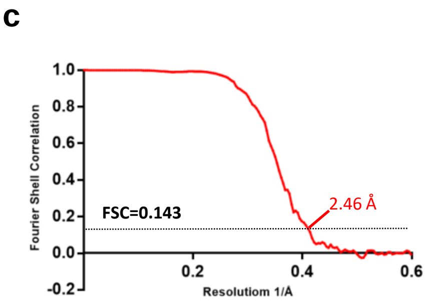

Figure supplement 4: Cryo-EM data processing scheme of the TRPML1 sample prepared in the presence of PI(4,5)P_2. (a) Representative micrograph. (b) Flow chart of the cryo-EM data processing procedure and the Euler angle distribution of particles used in the final three-dimensional reconstruction. Selected 2D class averages are shown. The final structure represent an open state. (c) Fourier Shell Correlation curves showing the overall resolution at FSC=0.143.

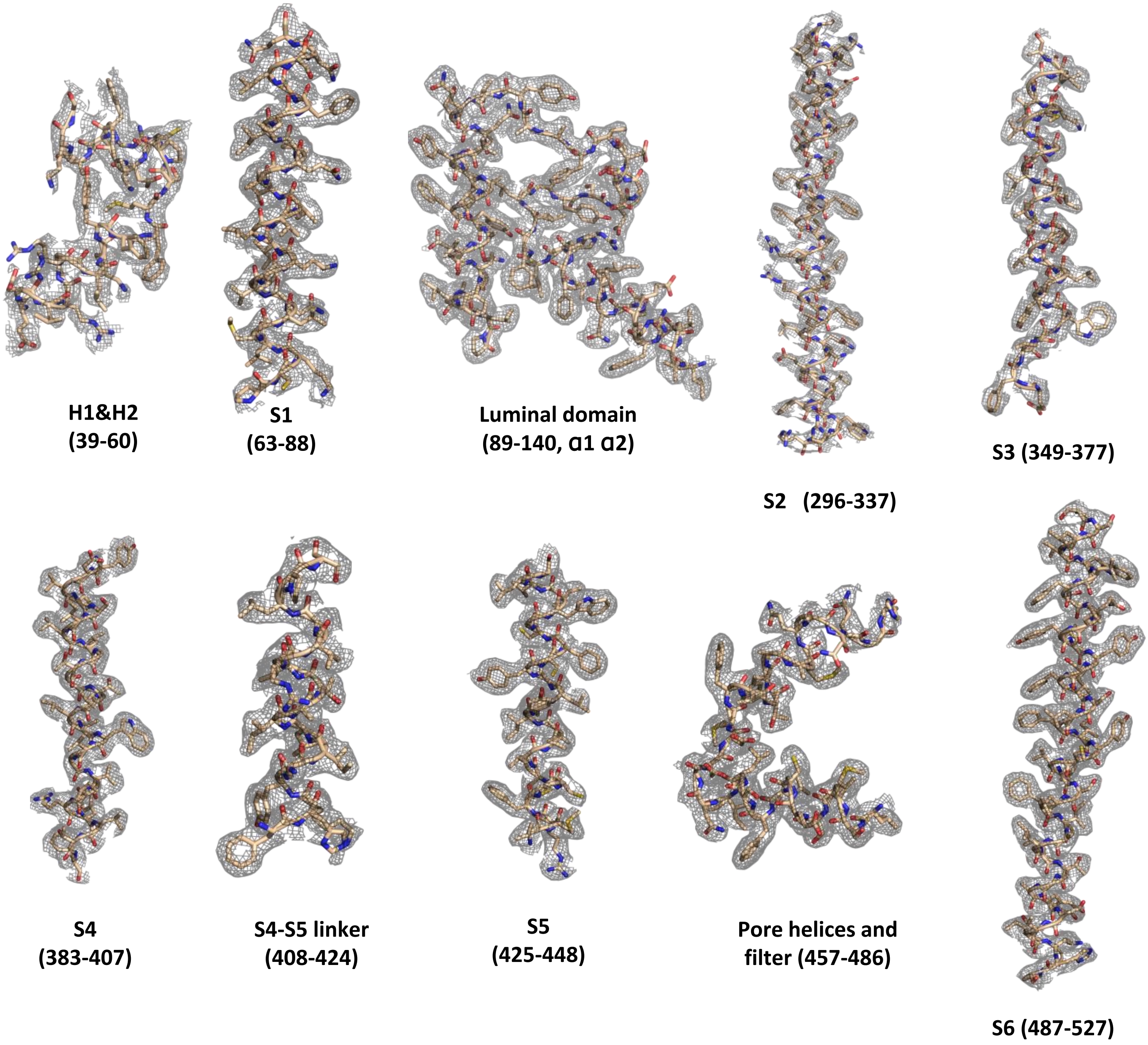

Figure supplement 5 : Sample density maps of the PI(4,5)P_2-bound closed TRPML1 structure contoured at 4 σ.

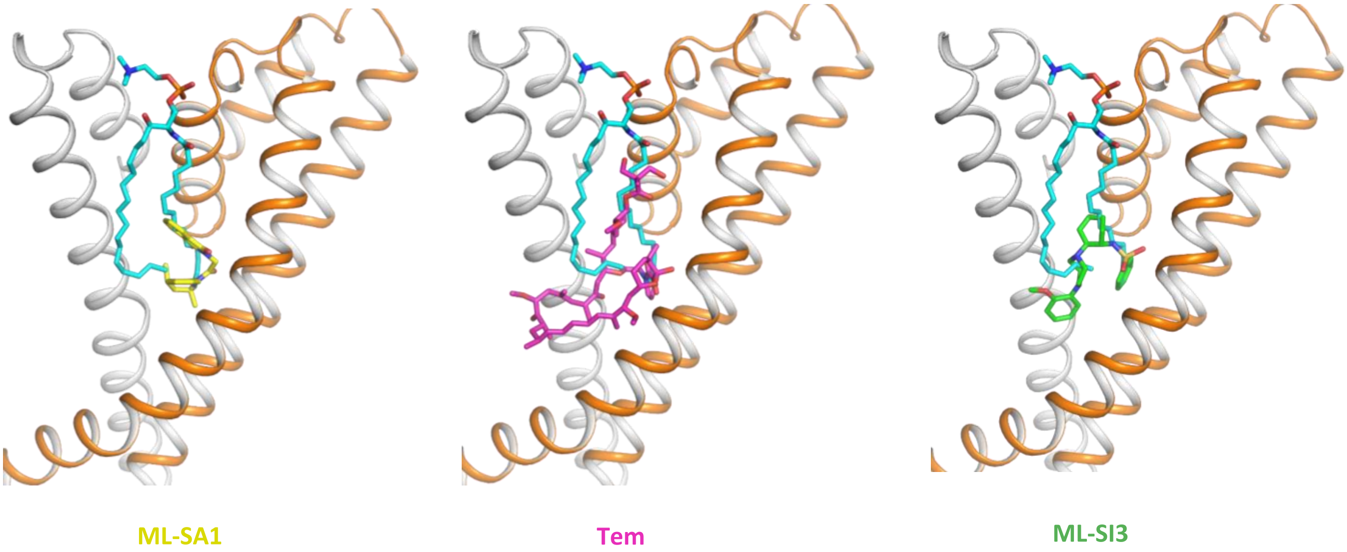

Figure supplement 6: Sphingomyelin (cyan) binding overlaps with that of agonist ML-SA1 (yellow), rapamycin analog Tem (magenta), or antagonist ML-SI3 (green).

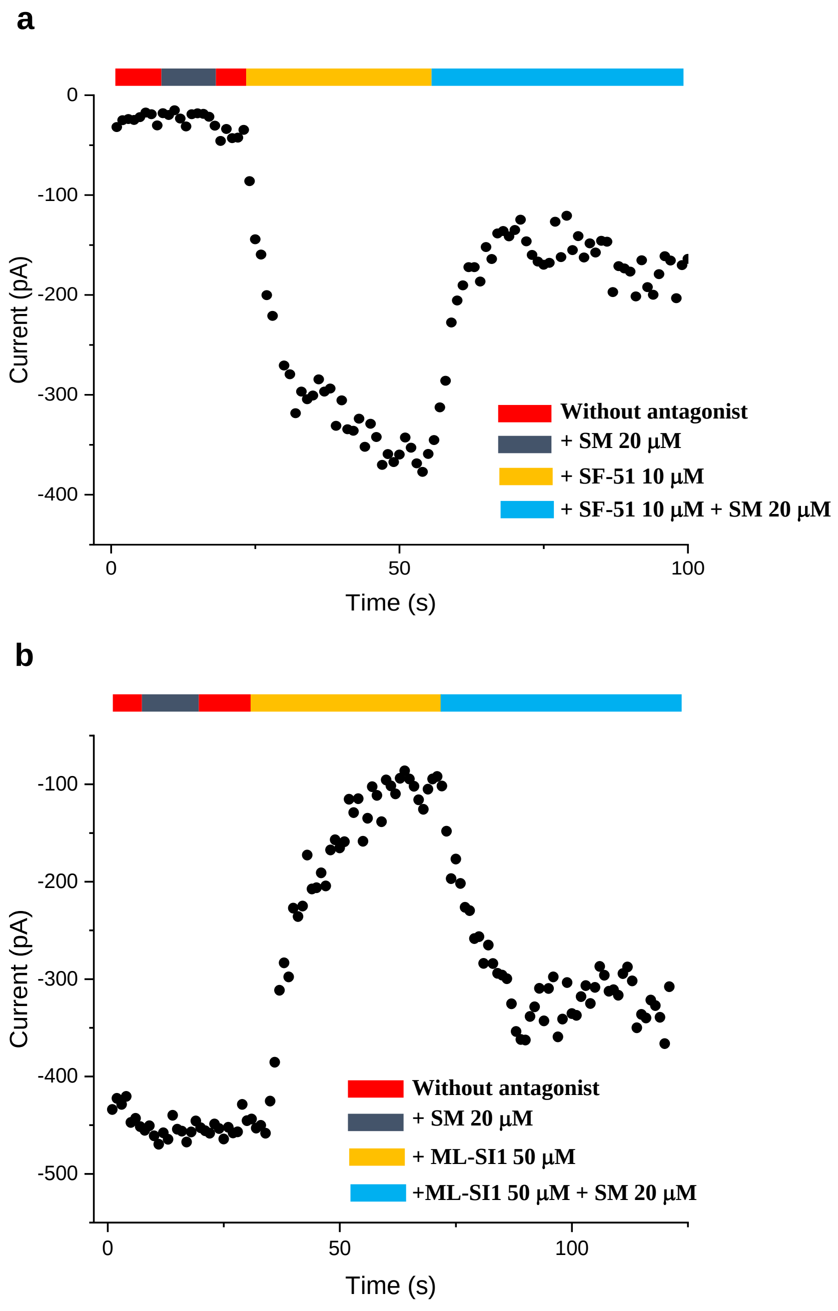

Figure supplement 7: Time course plots of sphingomyelin affected TRPML1 current amplitudes. (a) Sphingomyelin inhibition effect on SF-51-activated wild-type TRPML1. (b) SM activation effect on ML-SI1-inhibited Y404W mutant. Currents shown in (a) and (b) were recorded at -140mV using patch clamp in whole-cell configuration with pH 4.6 in the bath solution as the adverse effect of SM on agonist or antagonist is subtle and is measurable only at low luminal pH.

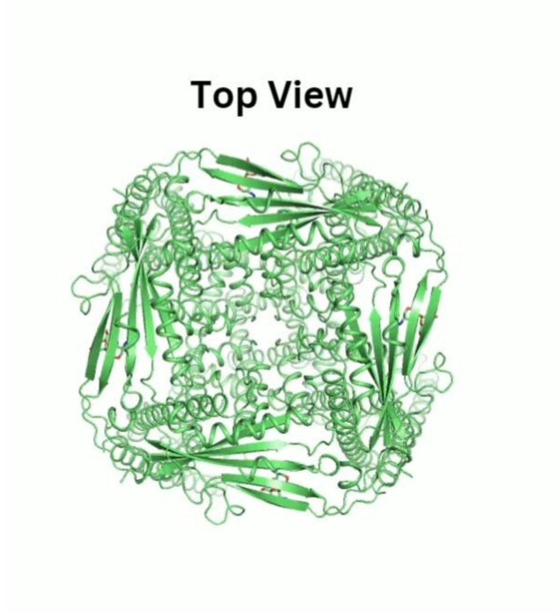

Movie supplement 1. Conformational changes between open and closed TRPML1
