# Supplementary material for: TRPML1 gating modulation by allosteric mutations and lipids (Design of allosteric mutations that recapitulate the gating of TRPML1 )
Source: bioRxiv. 2024 Jul 6:2024.07.04.602033. Preprint. [Version 1] doi: 10.1101/2024.07.04.602033 (PMC11245044; doi:10.1101/2024.07.04.602033)
Supplement: Supplementary file 1 [file NIHPP2024.07.04.602033v1-supplement-1.pdf]

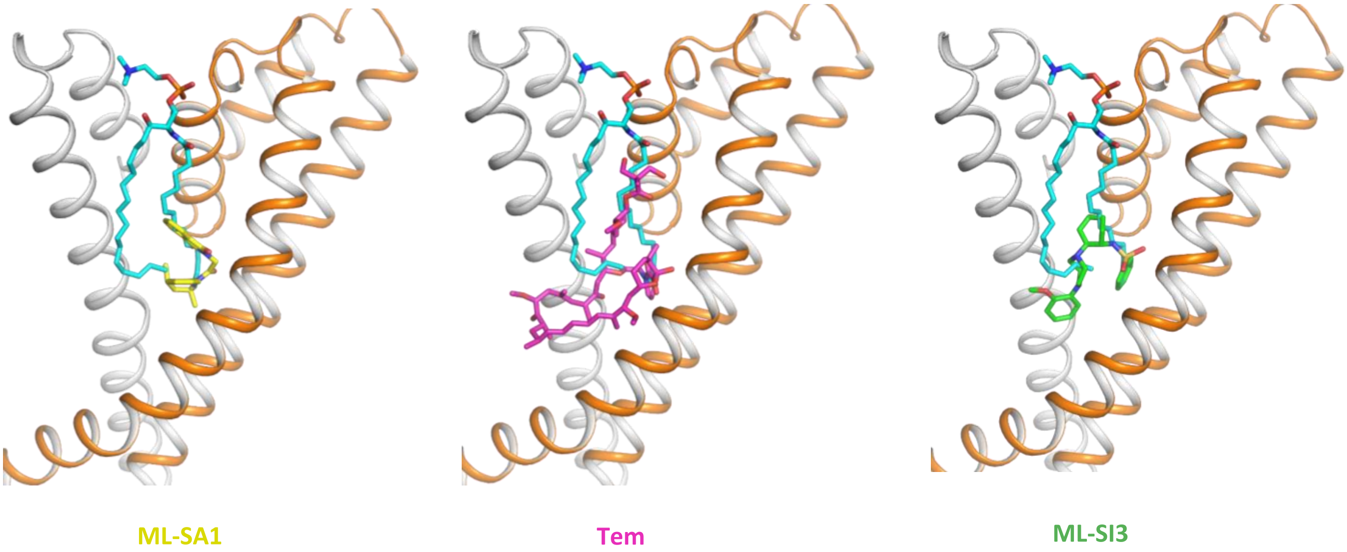

Figure supplement 1. Sphingomyelin (cyan) binding overlaps with that of agonist ML-SA1 (yellow), rapamycin analog Tem (magenta), or antagonist ML-SI3 (green).

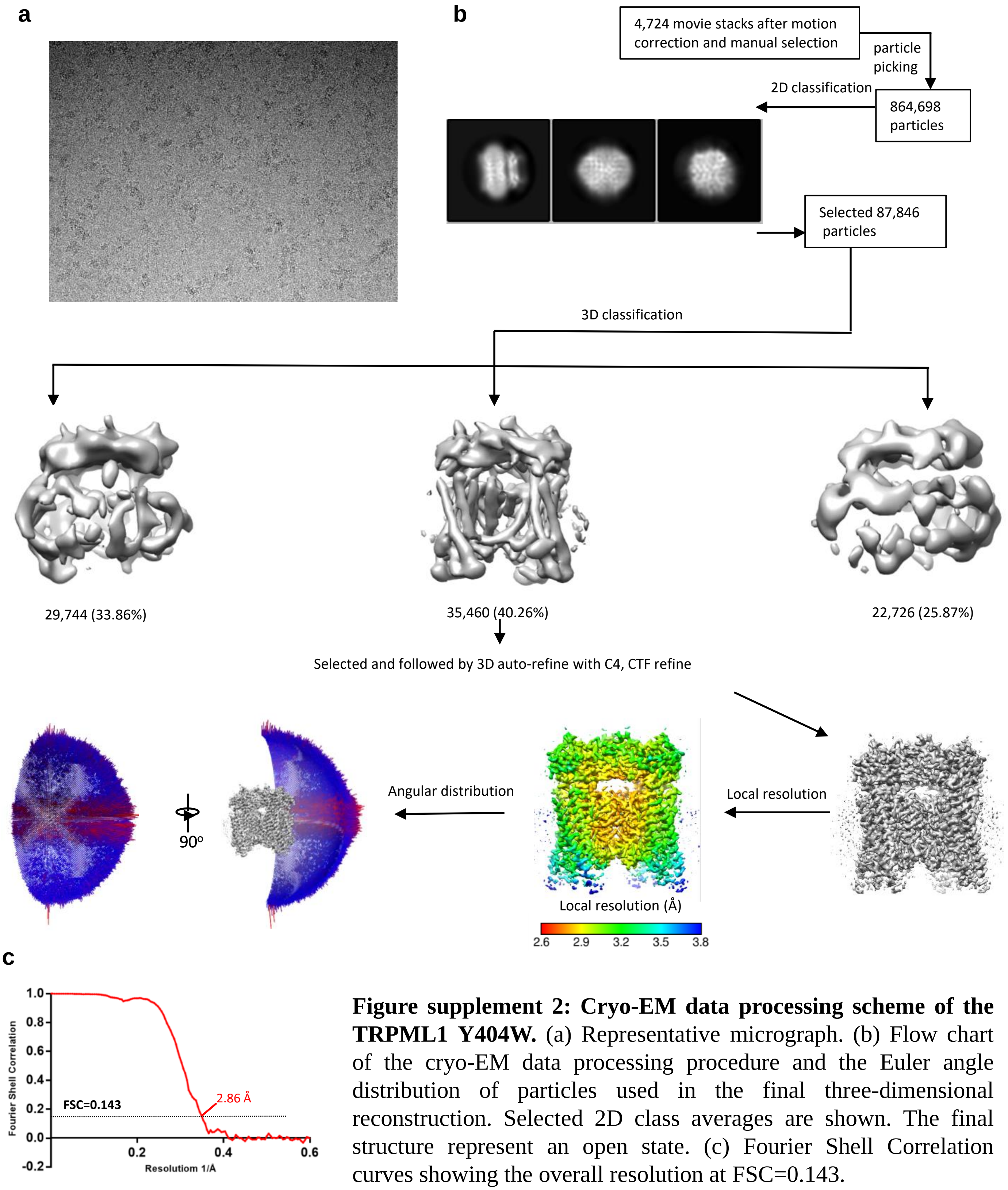

Figure supplement 2: Cryo-EM data processing scheme of the TRPML1 Y404W. (a) Representative micrograph. (b) Flow chart of the cryo-EM data processing procedure and the Euler angle distribution of particles used in the final three-dimensional reconstruction. Selected 2D class averages are shown. The final structure represent an open state. (c) Fourier Shell Correlation curves showing the overall resolution at FSC=0.143.

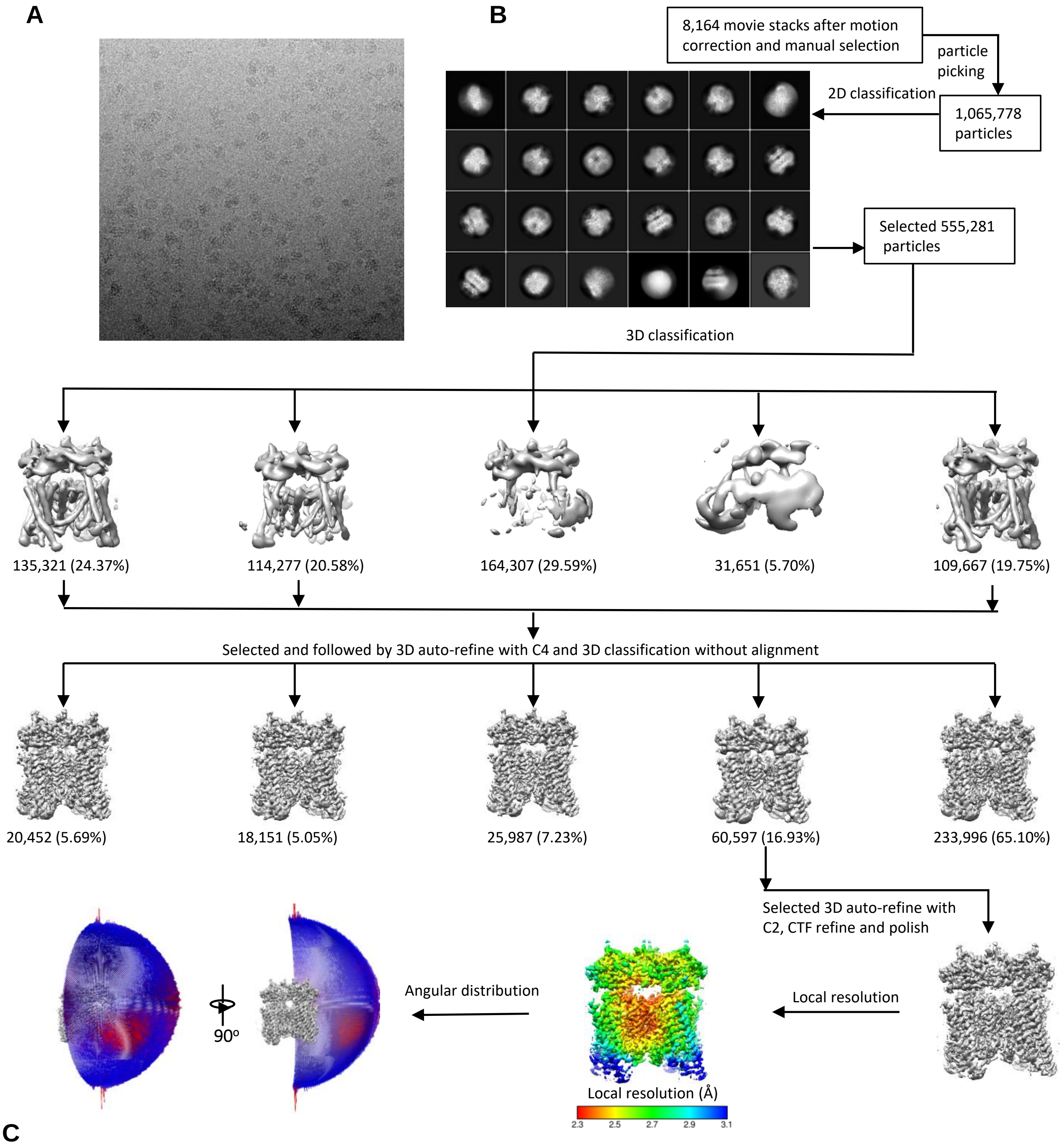

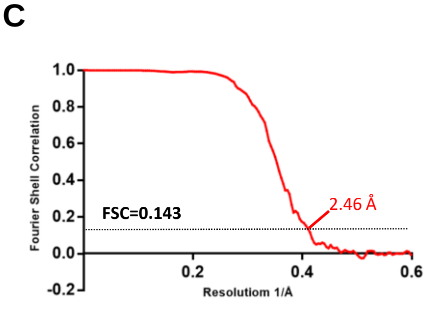

Figure supplement 3: Cryo-EM data processing scheme of the TRPML1 sample prepared in the presence of PI(4,5)P_2. (a) Representative micrograph. (b) Flow chart of the cryo-EM data processing procedure and the Euler angle distribution of particles used in the final three-dimensional reconstruction. Selected 2D class averages are shown. The final structure represent an open state. (c) Fourier Shell Correlation curves showing the overall resolution at FSC=0.143.

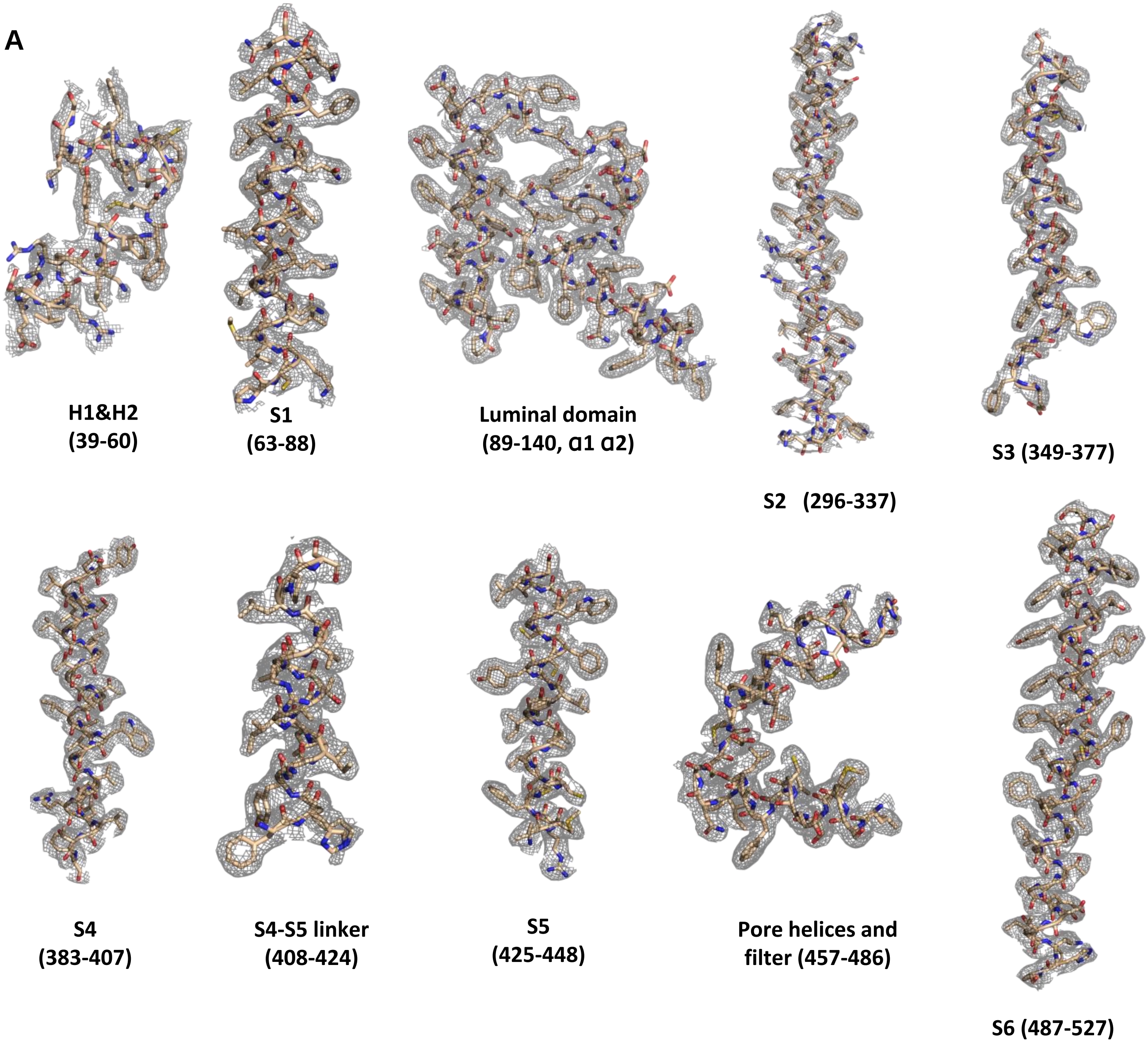

Figure supplement 4 : Sample density maps of the PI(4,5)P_2-bound closed TRPML1 structure contoured at 4 σ.

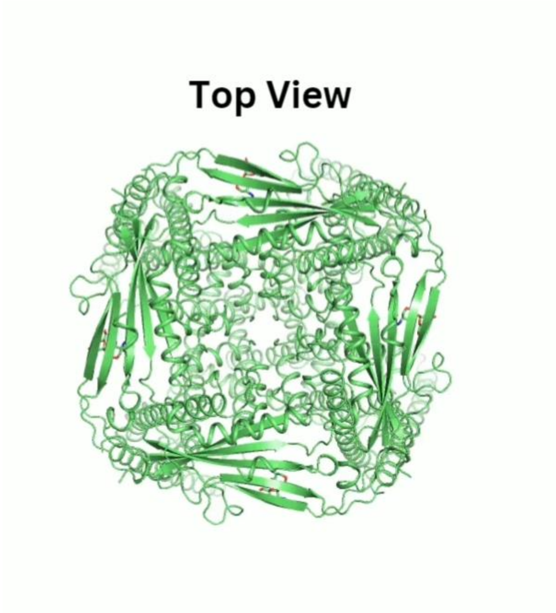

Movie supplement 1. Conformational changes between open and closed TRPML1
